# Prognostic value of neutrophile-to-lymphocyte ratio (NLR) and lactate dehydrogenase (LDH) levels for geriatric patients with COVID-19

**DOI:** 10.1186/s12877-022-03059-7

**Published:** 2022-04-25

**Authors:** Uğur Önal, Muhammet Gülhan, Neşe Demirci, Ahmet Özden, Nazlı Erol, Sema Işık, Sedat Gülten, Fatma Atalay, Nilay Çöplü

**Affiliations:** 1 Department of Infectious Diseases and Clinical Microbiology, Kastamonu Training and Research Hospital, Kastamonu, Turkey; 2 Department of Infectious Diseases and Clinical Microbiology, Uludag University, Faculty of Medicine, Bursa, Turkey; 3 Department of Radiology, Kastamonu Training and Research Hospital, Kastamonu, Turkey; 4 Department of Pulmonary Diseases, Kastamonu Training and Research Hospital, Kastamonu, Turkey; 5 Department of Internal Medicine, Kastamonu Training and Research Hospital, Kastamonu, Turkey; 6 Department of Biochemistry, Kastamonu Training and Research Hospital, Kastamonu, Turkey; 7 Department of Ear, Nose and Throat, Kastamonu Training and Research Hospital, Kastamonu, Turkey; 8 Department of Microbiology, Kastamonu Training and Research Hospital, Kastamonu, Turkey

**Keywords:** Prognostic factors, Geriatric patients, Covid-19, Neutrophile-to-lymphocyte ratio, lactate dehydrogenase

## Abstract

**Aim:**

In this study it was aimed to evaluate the prognostic factors for the geriatric patients with confirmed COVID-19 in a tertiary-care hospital at Kastamonu region of Turkey.

**Method:**

Patients (≥65-year-old) who had PCR positivity for COVID-19 between March 2020 and April 2020 in our center were recorded retrospectively. A *p* value less than 0.05 was considered significant. Ethical committee approval was given from the Bolu University with decision number 2020/176.

**Results:**

There were a total of 100 patients (44% female). In-hospital mortality was recorded as 7%. In univariate analysis for 1 month mortality, diabetes mellitus (*p* = 0.038), leucocyte count (*p* = 0.005), neutrophile count (*p* = 0.02), neutrophile-to-lymphocyte ratio (NLR) (*p* < 0.001), thrombocyte-to-lymphocyte ratio (TLR) (*p* = 0.001), C-reactive protein (CRP) (*p* = 0.002), lactate dehydrogenase (LDH) (*p* = 0.001), sequential organ failure assessment (SOFA) score (*p* = 0.001) and qSOFA score (*p* = 0.002) were found as independent risk factors. On admission, one point increase of NLR (*p* = 0.014, odds ratio (OR) = 1.371, 95% CI = 1.067–1.761) and one point increase of LDH (*p* = 0.047, OR = 1.011, 95% CI = 1.001–1.023) were associated with mortality on day 30 according to logistic regression analysis. The cut-off values were found as > 7.8 for NLR (83.33% sensitivity, 97.7% specificity) and > 300 U/L for LDH (100% sensitivity, 79.31% specificity) regarding the prediction of 30-day mortality.

**Conclusion:**

In order to improve clinical management and identify the geriatric patients with COVID-19 who have high risk for mortality, NLR and LDH levels on admission might be useful prognostic tools.

## Introduction

In December 2019, pneumonia cases with unknown origin were identified in Wuhan and a novel betacoronavirus (SARS-CoV-2) was identified from the respiratory tract speciemens of the affected patients [1]. The outbreak of the coronavirus disease (COVID-19) caused by SARS-CoV-2 was declared as a global pandemic by the World Health Organization in March 2020 [2].

Identifying the predictors of mortality in COVID-19 patients may help the clinicians in order to determine the optimal approach. In addition to several laboratory parameters, various independent risk factors were identified for the mortality in COVID-19 patients such as advanced age, male gender, current smoking status, comorbidities, sepsis and septic shock [3].

Overall risk of mortality for COVID-19 patients were higher in older patients. In addition, clinical signs of respiratory failure or laboratory biomarkers showing inflammation such as decrease in lymphocyte count with high neutrophile and lactate dehydrogenase (LDH) levels were associated with mortality in COVID-19 patients [4].

Elderly people with comorbidities including diabetes mellitus or hypertension are expected to be more susceptible to COVID-19 regarding the disease severity or mortality and the previous studies were mainly focused on general population regarding the evaluation of prognostic factors for COVID-19 patients [3, 4]. Vakili et al. reviewed the laboratory findings in COVID-19 patients for different age groups and lymphopenia, elevated C-reactive protein (CRP) or LDH levels were found as significantly higher in elderly patients compared to young and middle-aged groups [5].

Although the COVID-19 pandemic is a global healthcare problem for all age groups, there is an urgent need to define the prognostic factors for the patients with COVID-19, especially in geriatric age group since the older age is already associated with poor prognosis.

Herein, we aimed to evaluate the prognostic factors for the geriatric patients with confirmed COVID-19 in a tertiary-care hospital at Kastamonu region of Turkey.

## Material and methods

Between the dates of 20th March 2020 and 30th April 2020, geriatric patients (≥65-year-old) who had polymerase chain reaction (PCR) positivity for COVID-19 were recorded in our center, retrospectively. The patients were evaluated following the hospitalization. Crude mortality (30-day) was recorded in this study.

Sequential Organ Failure Assessment (SOFA) score was calculated by the parameters (0 to 4 points per each parameter) of Glasgow Coma Scale, PaO2/FiO2 with respiratory support, thrombocyte count, bilirubin level, serum creatinine with urine output, mean arterial pressure and the use of vasopressor agents whereas quick SOFA (qSOFA) score was calculated by the parameters (1 point for the presence of each parameter) of respiratory rate ≥ 22 breaths/min, systolic blood pressure ≤ 100 mmHg and altered mental state [6].

Case assessment forms included data related to demographical, clinical and laboratory findings of COVID-19 patients.

### Inclusion criteria were

- Meeting the criteria of clinical spectrum of SARS-CoV-2 infection according to National Institutes of Health (NIH) COVID-19 Treatment Guidelines [7]
- Positive PCR result for COVID-19 (only patients with confirmed diagnosis were included)

### Exclusion criteria were

- Age < 65-year-old (only geriatric patients were included)

### Microbiological analysis

COVID-19 RT-qPCR (Bio-Speedy, Bioeksen, TR) detection kits and C1000 Touch Termal Cycler CFX96 Real Time System (Biorad, UK) were performed for the diagnosis.

### Ethics

The study was approved by Ethical committee of Bolu University. Ethical committee approval was given from the Bolu University with decision number 2020/176. Also, the permission from Republic of Turkey, Ministry of Health was given.

### Statistical analysis

SPSS 25.0 program (Statistical package for the social sciences) was used for the statistical analysis. Comparison of categorical values between the two groups was performed via Chi-square test. Independent sample t-test was performed for the normally distributed numerical values of the independent groups whereas Mann-Whitney U test was performed for ordinal or continuous values which were distributed not normally.

Univariate and binary logistic regression analysis were performed using the enter method. Mortality was the dependent variable and the variables with a *p* < 0.05 in univariate analysis as covariates. A *p* value of less than 0.05 was considered significant. ROC analysis was performed by using MedCalc statistical software program.

## Results

### General characteristics

There were a total of 100 patients (44% female) fulfilling the study inclusion criteria. Mean age was recorded as 72.14 ± 0.55 years. Mean leukocyte, neutrophile, lymphocyte, thrombocyte levels at the hospitalization were 6243 ± 299/mm^3^, 3821 ± 273/mm^3^, 1735 ± 86/mm^3^ and 270,451 ± 11,170/mm^3^, respectively. Mean values of the acute phase reactants such as ferritin, CRP and procalcitonin (PCT) were recorded as 254.47 ± 35.64 μg/L, 43.52 ± 8.11 mg/L and 0.29 ± 0.17 μg/L, respectively. Mean SOFA and qSOFA scores were recorded as 0.88 ± 0.17 and 0.15 ± 0.05. In addition to this, mean D-dimer and LDH levels were recorded as 1.59 ± 0.27 mg/L and 268.17 ± 11.81 U/L.

The most common symptoms at the admission were cough (63%), fever (62%) and dyspnea (33%) whereas the most common comorbidities were hypertension (18%) and diabetes mellitus (14%).

Mean neutrophil-to-lymphocyte ratio (NLR) and thrombocyte-to-lymphocyte ratio (TLR) at the hospitalization were recorded as 3.34 ± 0.48, 208.84 ± 17.56.

### Mortality and associated factors

Overall 30-day mortality was 7%. Mortality rate was higher for men than women but the difference was non-significant (5/56 vs 2/44, *p* = 0.402). Also, mortality rates increased with age but there was no statistically significant difference (73.14 ± 0.50 vs 72.13 ± 0.59, *p* = 0.206).

In univariate analysis for 30-day mortality, diabetes mellitus (*p* = 0.038), leukocyte count (10.971 ± 2.190/mm^3^ vs 5917 ± 252/mm^3^, *p* = 0.005), neutrophile count (9268 ± 1563/mm^3^ vs. 3445 ± 224/mm^3^, *p* = 0.02), NLR (15.24 ± 4.58 vs. 2.52 ± 0.26, *p* < 0.001), TLR (552.82 ± 127.44 vs. 185.12 ± 13.65, *p* = 0.001), CRP (210.66 ± 62.35 mg/L vs. 31.99 ± 5.97 mg/L, *p* = 0.002), LDH (468.33 ± 49.26 U/L vs. 254.36 ± 10.75 U/L, *p* = 0.001), SOFA score (4.57 ± 1.36 vs 0.6 ± 0.11, *p* = 0.001) and qSOFA score (1.29 ± 0.47 vs 0.06 ± 0.02, *p* = 0.002) were found as independent risk factors (Table 1).

**Table 1:** Univariate analysis of independent variables on mortality (30-day)

Risk Factors (On admission)		Mortality		***P*** value
		Yes	No	
**Age**	years	73.14 ± 0.50	72.13 ± 0.59	*p* = 0.206
**Gender**	Male (n, %) Female (n, %)	5 (9%) 2 (5%)	51 (91%) 42 (95%)	*p* = 0.402
**Hypertension**	Present (n, %) Absent (n, %)	3 (17%) 4 (5%)	15 (83%) 78 (95%)	*p* = 0.09
**Diabetes mellitus**	Present (n, %) Absent (n, %)	3 (21%) 4 (5%)	11 (79%) 82 (95%)	***p*** = 0.038*
**CRP**	mg/L	210.66 ± 62.35	31.99 ± 5.97	***p*** = 0.002*
**Procalcitonin**	ug/L	1.02 ± 0.26	0.26 ± 0.18	*p* = 0.431
**Ferritin**	ug/L	840.16 ± 251.25	214.07 ± 30.10	*p* = 0.055
**Leukocyte count**	/mm^3^	10.971 ± 2.190	5917 ± 252	***p*** = 0.005*
**Neutrophile count**	/mm^3^	9268 ± 1563	3445 ± 224	***p*** = 0.02*
**Lymphocyte count**	/mm^3^	1250 ± 729	1768 ± 79	*p* = 0.135
**Thrombocyte count**	/mm^3^	302.166 ± 29.568	268.264 ± 11.761	*p* = 0.458
**NLR**		15.24 ± 4.58	2.52 ± 0.26	***p*** < 0.001*
**TLR**		552.82 ± 127.44	185.12 ± 13.65	***p*** = 0.001*
**LDH**	U/L	468.33 ± 49.26	254.36 ± 10.75	***p*** = 0.001*
**D-dimer**	mg/L	3.87 ± 0.7	1.43 ± 0.28	*p* = 0.086
**SOFA**		4.57 ± 1.36	0.6 ± 0.11	***p*** = 0.001*
**qSOFA**		1.29 ± 0.47	0.06 ± 0.02	***p*** = 0.002*

*: *p* < 0.05

### Logistic regression analysis for mortality

In logistic regression analysis diabetes mellitus, CRP, leucocyte count, neutrophile count, NLR, TLR, LDH, SOFA and qSOFA were analysed.

On admission, one point increase of NLR (*p* = 0.014, odds ratio (OR) = 1.371, 95% CI = 1.067–1.761) and one point increase of LDH (*p* = 0.047, odds ratio (OR) = 1.011, 95% CI = 1.001–1.023) were associated with mortality according to logistic regression analysis (Table 2).

**Table 2:** Results of logistic regression analysis

Covariate (On admission)	Odds ratio (OR)	95% Confidence interval (CI)	***P*** value
**One point increase of NLR**	1.371	1.067–1.761	**0.014**
**One point increase of LDH**	1.011	1.001–1.023	**0.047**

### Receiver operating characteristic (ROC) curve analysis of prognostic factors for 30-day mortality

Prognostic factors on the hospitalization such as NLR, LDH, CRP, SOFA and qSOFA were analyzed via ROC curve analysis in terms of 30-day mortality prediction. The highest area under curve (AUC) value was recorded for NLR with AUC: 0.944 (*p* < 0.0001, 95% CI: 0.877–0.981, Youden Index (YI): 0.8103) which was followed by LDH as AUC: 0.927 (*p* < 0.0001, 95% CI: 0.854–0.971, YI: 0.7931), CRP as AUC: 0.899 (*p* < 0.0001, 95% CI: 0.820–0.952, YI:0.7874), SOFA as AUC: 0.916 (*p* < 0.0001, 95% CI: 0.843–0.962, YI: 0.6713) and qSOFA as AUC: 0.834 (*p* = 0.0004, 95% CI: 0.746–0.901, YI: 0.6498). Regarding the prediction of 30-day mortality, the optimal cut-off values were found as > 7.8 for NLR with 83.33% sensitivity, 97.7% specificity and > 300 U/L for LDH with 100% sensitivity, 79.31% specificity, respectively.

## Discussion

In order to improve clinical management of the patients with COVID-19 who have high risk for mortality, we believe that it is necessary to define the prognostic parameters especially for geriatric age group. This study emphasizes that simple complete blood count analysis and/or lactate dedydrogenase levels can be used for the idendify the high risk geriatric patients.

The NLR which was simply calculated by dividing neutrophile count/lymphocyte count is an easily available tool especially for developing countries and it can be used as a biomarker for the prognosis of various cardiovascular diseases such as myocardial infarction, heath failure and atherosclerosis [8]. COVID-19 was associated with systemic inflammation and hypercoagulability which can also be accompanied by cytokine storm that alters the hematological parameters. Thus, the link between COVID-19 and cardiovascular diseases was investigated by several studies and NLR might be used as an independent prognostic marker for COVID-19 in terms of disease severity [9]. In addition to this, NLR had also been evaluated for the septic patients that it can be useful as a prognostic parameter via having a correlation with mortality prediction scores such as APACHE-II and SOFA [10].

Mahat et al. demonstrated that higher levels of CRP, PCT, IL-6, ferritin, and NLR for the patients with mortality via a meta-analysis of 83 studies in COVID-19 patients [11]. Similar to that, another meta analysis also investigated a total number of 6033 patients with COVID-19 in terms of mortality and the study showed that the pooled mortality risk ratio in patients with elevated versus normal levels of NLR was 2.74 (95% CI: 0.98–7.66), regardless of the different cut-off values [12]. NLR might be used as both prognostic and diagnostic purposes. A retrospective study with a total number of 233 patients admitted to emergency department for COVID-19 revealed that CRP (*p* < 0.001), lactate dehydrogenase (LDH) (*p* = 0.038), NLR (*p* = 0.001) and PLR (*p* < 0.001) levels were found as significantly higher in COVID-19 positive patients [13]. On the other hand, parallel to our findings, Bağ Soytaş et al. also investigated a total number of 218 geriatric patients (≥65-year-old) with COVID-19 and they found that NLR (OR = 1.097, 95% CI: 1.012–1.188, *p* = 0.025) and LDH (OR = 1.002, 95% CI: 1.001–1.004, *p* = 0.003) values on admission as independent risk factors for the mortality [14]. Kalyon et al. also showed that NLR can be a useful prognostic tool in order to determine the high risk of geriatric patients with COVID-19 in terms of mortality and they found the cut-off of NLR as 4.02 (sensitivity, 74.14%; sensitivity, 65.81%) with an area under the ROC curve as 71.7% (*p* < 0.001) [15]. However, there is no optimal cut-off value of NLR for the predicting mortality in COVID-19 patients. Several studies revealed values with a range of 7.9 to 11.8 [16, 17]. In our study, we also found that NLR had the higher AUC value than LDH, CRP, SOFA and qSOFA with a cut-off level as 7.8 (83.33% sensitivity, 97.7% specificity) in terms of predicting the mortality in COVID-19 patients. The possible explanations for these differences might be due to different demographical data or different clinical presentation of the patients in terms of disease severity.

Similar well-known mechanism to COVID-19 disease, acute tissue hypoxia with inflammation results the increase of LDH levels in human immunodeficiency virus infected patients with *Pneumocystis carinii* pneumonia [18]. Li et al. investigated a total number of 203 patients with COVID-19 and they showed that elevated LDH level at admission is an independent risk factor for the disease severity (Hazard Ratio (HR): 2.73, 95% CI: 1.25–5.97; *p* = 0.012) and mortality (HR: 40.50, 95% CI: 3.65–449.28; *p* = 0.003) [19]. They also revealed a cut-off level for LDH as 359.50 U/L with 93.8% sensitivity, 88.2% specificity for predicting death [19]. A cohort study in oldest-old geriatric population (80 years or older) with COVID-19 also revealed that serum LDH levels were associated with severe disease (*p* = 0.013; OR 2.55; 95% CI: 1. 21-5.37) [20]. Similar to this, De Smet et al. also showed that baseline LDH level was found as an independent risk factor for mortality in geriatric patients with COVID-19 (OR:1.005, 95% CI: 1.000–1.011) [21]. In our study, we also found that increase of LDH level predicts the higher mortality in COVID-19 patients and as a cut-off level > 300 U/L (100% sensitivity, 79.31% specificity) might be *discriminative.*

*Our study has several limitations. First of all, we conducted a retrospective analysis between the dates of March and April 2020. The virus had many mutations during the last months, therefore, there might be some differences regarding the prognosis of disease. Secondly, we investigated the all cause mortality due to lack of the autopsy procedure.*

## Conclusion

Among the geriatric patients with COVID-19 who have high risk for mortality, we found that NLR and LDH levels on admission might be useful prognostic factors. In addition to this, the optimal cut-off values were found as > 7.8 for NLR (83.33% sensitivity, 97.7% specificity) and > 300 U/L for LDH (100% sensitivity, 79.31% specificity) regarding the prediction of 30-day mortality.

## Data Availability

The datasets generated and/or analysed during the current study are not publicly available due to ethical/legal/commercial reasons but are available from the corresponding author on reasonable request.
